# Metallothionein in Brain Disorders

**DOI:** 10.1155/2017/5828056

**Published:** 2017-09-20

**Authors:** Daniel Juárez-Rebollar, Camilo Rios, Concepción Nava-Ruíz, Marisela Méndez-Armenta

**Affiliations:** ^1^Laboratorio de Neuropatología Experimental, Instituto Nacional de Neurología y Neurocirugía MVS, Mexico City, Mexico; ^2^Departamento de Neuroquímica, Instituto Nacional de Neurología y Neurocirugía MVS, Mexico City, Mexico

## Abstract

Metallothioneins are a family of proteins which are able to bind metals intracellularly, so their main function is to regulate the cellular metabolism of essential metals. There are 4 major isoforms of MTs (I–IV), three of which have been localized in the central nervous system. MT-I and MT-II have been localized in the spinal cord and brain, mainly in astrocytes, whereas MT-III has been found mainly in neurons. MT-I and MT-II have been considered polyvalent proteins whose main function is to maintain cellular homeostasis of essential metals such as zinc and copper, but other functions have also been considered: detoxification of heavy metals, regulation of gene expression, processes of inflammation, and protection against free radicals generated by oxidative stress. On the other hand, the MT-III has been related in events of pathogenesis of neurodegenerative diseases such as Parkinson and Alzheimer. Likewise, the participation of MTs in other neurological disorders has also been reported. This review shows recent evidence about the role of MT in the central nervous system and its possible role in neurodegenerative diseases as well as in brain disorders.

## 1. Introduction

In 1957, the metallothionein was first identified by Margoshes and Vallee [1] in the cortex of horse kidney. This protein was later characterized biochemically by Kagi and Valle [2] in 1960, since then it has been widely studied in several aspects. This family of proteins is characterized by elevated heavy metal and cysteine content, so it was named metallothionein (MT). Different functions have been described for this protein, among them are storage and transport of essential metals such as copper and zinc, angiogenesis processes, protection against reactive oxygen species (ROS) and DNA damage, gene regulation, inflammatory processes, and cell survival among others [3, 4]; also the MTs have been implicated in processes of neuroprotection and neuroregeneration. The scientific interest in the functional role of MT has been increasing as more physiological functions have been described, and MT is now considered to be protein protecting against oxygen radicals and oxidative damage to the central nervous system; although MT was discovered over 60 years ago, its physiological functions are still unclear in the brain disorders. In this brief review, we focus mainly on emphasizing the physiological role of MT in brain disorders.

## 2. Classification and Biochemical Properties of MT

**Figure 1:**
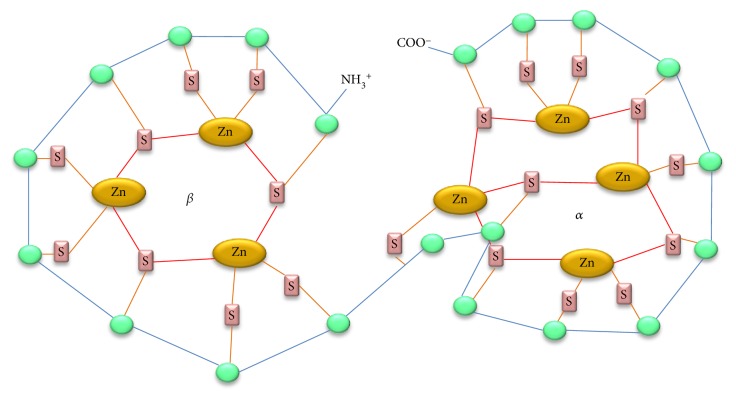
Schematic representation of the MT structure. The metal-thiolate complexes (with C-terminal domain of the *α*- and N-terminal chain in the *β* chain); the green ovals represent the 20 cysteine residues bound to the sulfur compound (S) which binds divalent to zinc, three bonds to zinc of the *β* chain and four to the *α* chain by adding seven divalent bonds.

MT is a family of proteins characterized by their low molecular weight, they have a single peptide chain containing 61–68 amino acids, 20 of them are cysteines distributed in two domains *α* and *β*-clusters [5, 6], and they are bound in total 7 ions of divalent metals. The single polypeptide chain of MT has the structure cys-x-cys, cys-xy-cys, and cys-cys where the x and y represent noncysteine amino acids; the stoichiometric form of the protein shows 7 ions for each 20 cysteines (Figure 1) forming metal-thiolate complexes, enabling the MT to bind to 7–10 g of metal/mol MT atoms [7–10]. The binding of MT to metals is through the thiol group (SH) found in cysteine residues; the metal-free protein named as apo-metallothionein or thionein has a predominantly disordered structure, which makes it highly vulnerable to proteolytic processes [11]. Normally, MT has a high affinity for divalent essential metals such as Cu and Zn, although it also has shown a high affinity for toxic metals like cadmium and mercury giving rise to metal-thiolate clusters [12]. Up to now, 4 isoforms of the protein are recognized, which are structurally homologous and enough conserved during evolution [13].

The existence of four MT genes in a region of 50 kb on chromosome 8 has been reported in mice, whereas that in humans, the MT genes have been found on chromosome 16q13 which are encoded by a group of closely related multigene; the presence of seven MT-I functional genes: A, B, C, D, E, F, G, H, and X have been described, which represent microheterogeneity of the MT-I protein as well as a single gene encoding the other isoform of MT-II (MT-IIA), MT-III, and MT-IV [6, 14]. The isoforms of MT-I and MT-II are predominantly expressed in various animal tissue; MT-III and MT-IV are isoforms that are expressed in more specific tissues.

## 3. Expression and Regulation of MT Genes

**Figure 2:**
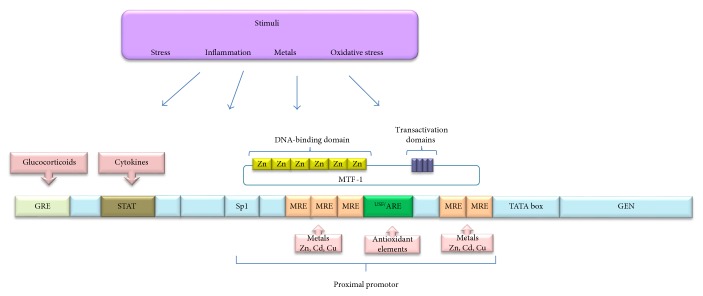
Schematic representation of MT-I and MT–II gen. The cis-acting response elements: glucocorticoids responsive elements (GREs), metal responsive elements (MREs), and antioxidant responsive elements (AREs). Signal transducter and activator of transcription (STAT) Sp1-binding site (Sp1), upstream stimulatory factor (USF), and metalloregulatory transcription factor with functional domains (MTF-1).

Expression of the MT-I and MT-II genes is achieved through two important elements present in the regulatory region of the gene: the TATA box (the core promoter element) and several cis-acting response elements, which are metal-responsive elements (MRE) and antioxidant-responsive elements (ARE). Both elements are mediated by the metal response element binding transcription factor (MTF-1), glucocorticoid responsive elements (GRE), signal transducer, and activator of transcription (STAT); the AREs can be downregulated positively or negatively for transcriptional factors such as Sp1, TPA, USF, Ap1, and Ap2 [9, 15, 16]. The expression of MT gene is significantly regulated by the Zn finger transcription factor MTF-1, which is a metalloregulatory protein, that binds to the proximal MER promoters, through their Zn fingers; the DNA binding activity is reversibly activated in response to changes in free intracellular Zn concentrations (Figure 2) [14, 16]. Similarly, previous studies have reported that linkers between the six different fingers participate actively in the modulation of MTF-1 translocation from the cytoplasm to the nucleus and its binding to the promoter of the MT-I gene; it should be noted that Zn is the only recognized metal that activates MTF-1; however, it has also been seen that this factor can be activated by oxidative stress [11, 16, 17]. It is commonly accepted that the expression of MT-I and MT-II is highly inducible in response to a wide variety of stimuli including metals, hormones, oxidative stress, and inflammatory mediators (such as cytokines); the synthesis of this protein is activated through the action of cis-acting DNA sequences in response to stimuli [9, 12, 14].

## 4. Physiology of MTs

**Figure 3:**
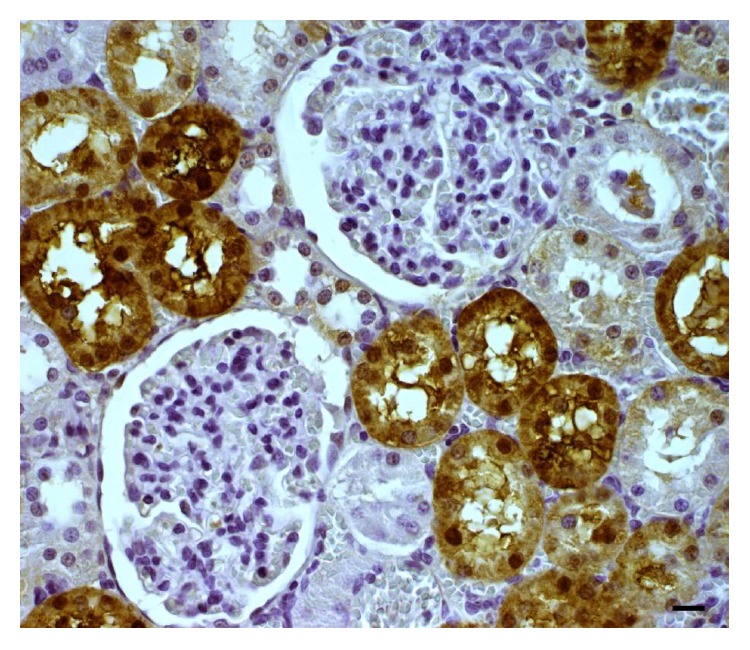
Representative images of immunohistochemical study of the presence of MT in rat kidney. The MT immunoreactivity (brown color) in the cells of the proximal and distal convoluted tubules in kidney. The image shows a glomerular corpuscle with the presence of Bowman's capsule surrounded by numerous contoured tubules. 1000x magnification.

Several papers have supported the cellular functions of MTs, which mainly include homeostasis and transport of essential metals (Zn and Cu), detoxification of toxic metals, apoptosis, modulation of intracellular redox balance, anti-inflammatory processes, free radical scavenging, and protection of neurons against neuronal lesions [18–20]. As mentioned above, MT was initially described as a protein responsible for the accumulation of cadmium in the kidneys of horses [15]. It is now known that this protein is mainly involved in the detoxification of heavy metals such as cadmium and mercury; these metals bind to the protein by sequestering them and decreasing the acute effects of these toxic metals [21]. The induction in the expression of MT by heavy metals and subsequent accumulation of metal in the cell has been used as a biomarker in the field of environmental toxicology (Figure 3) [12].

MT has as its main physiological function to maintain the homeostasis and transport of essential metals such as Zn and Cu. Zn is a physiologically important metal that provides structural and catalytic functions to a variety of proteins. The Apo-MT form is a Zn acceptor (which binds with high affinity), increasing intracellular Zn concentrations; when these Zn levels are insufficient to stabilize the protein, the MT is rapidly proteolyzed, so the Zn is released by the degradation of MT, causing the intracellular Zn to remain at a balanced concentration [14]. Traditionally, MT has been considered as an intracellular protein, is localized in cytoplasm, and by translocation can also be found in the nucleus; however, more recent reports suggest that MT can be localized in a variety of extracellular spaces [22].

MT is a vital protein in the cellular defense antioxidant system, and its protective role against ROS damage in biological systems has been widely reported. Different studies have shown that the thiolate ligands in cysteine residues confer the redox activity of MT; these residues can be oxidized by cellular oxidants, and during this process, Zn is released, causing a decrease in lipid peroxidation levels [23]. It is known that, when there is an increase in oxidative stress levels, MT is able to scavenge a variety of ROS including hydroxyl radicals and superoxide anion, hydrogen peroxide, radicals of reactive nitrogen species, and nitric oxide radicals [4, 6, 24, 25]. Compared with other antioxidants such as super oxide dismutase, catalase, and glutathione peroxidase, MT may be considered a more effective antioxidant [26, 27].

Also, there are reports suggesting an anti-inflammatory role of MT, which includes protection of the nervous system [28], in lung lesions, and acute hepatic injury [29]; it is proposed that this effect may be due to activation or inhibition of the expression of pro- and anti-inflammatory interleukins depending on the physiological conditions present [27, 30].

## 5. MT in the Central Nervous System

The brain is highly susceptible to oxidative stress due to the high levels of oxygen consumption, so that the role of MT as a protein that protects against these processes is very important; as mentioned above, maintaining intracellular Zn homeostasis is one of the main functions of this protein and the brain contains high levels of this metal hence showing the importance of MT in the central nervous system.

MT-I and MT-II are regulated in a coordinated manner; their biosynthesis can be induced by a diverse variety of stimuli such as stress conditions, glucocorticoid levels, cytokines, ROS, and metal ions, which have been reported to occur through various regions of the central nervous system (the cortex, brainstem, spinal cord, thalamus, hippocampus, basal forebrain, neocortex, cranial nerve nuclei, olfactory bulb, and cerebellum) [22, 31]. MTs are proteins predominantly expressed in the cellular cytoplasm, mitochondria and lysosomes, within astrocytes, cells, meningeal cells, ependymal cells, choroid, arachnoid, and pia mater [32, 33]. It has recently been reported that MT-I and MT-II are also distributed intra- and extracellularly, so neurons can incorporate these proteins into their cytoplasm through a family of low-density lipoprotein receptors (megalin) [34–36]. Although the cells of microglia, oligodendrocytes, and neurons commonly do not show MT-I or MT-II expression, it has been observed that in response to brain damage, the protein can be expressed [4, 30].

MT-III was isolated and reported for the first time in 1991 by Uchida et al. [37] in patients with Alzheimer's disease and was identified as a growth-inhibitory factor (GIF) or neuronal growth-inhibitory factor. Subsequent *in vitro* and *in vivo* studies revealed that MT-III isolated from human and bovine brains is predominantly expressed in neurons and contains 4 divalent metal ions of Cu and 3 or 4 divalent Zn metals, differing from the divalent Zn metals which are normally found in the MT-I and MT-II [12]. Another difference with respect to MT-I and MT-II is that MT-III does not appear to be regulated by metals or glucocorticoids, so it is not considered inducible and its neuroanatomical distribution is well related to areas with high concentrations of Zn [8]. In normal brain, expression of MT-III is more restricted; it has been reported that mRNA is related to neuronal cells, whereas MT-III upregulation can be observed in glial cells after brain damage [38]. Although there are contradictory reports about the expression of MT-III, it is generally accepted that the cellular localization of MT-III is mainly in neurons especially those with high concentrations of Zn, suggesting a specific function; none of the other MT isoforms have a growth-inhibitory activity suggesting that this is a specific functional characteristic of MT-III [30, 39–42].

The neuroinflammation process independently contributes to neuronal dysfunction as well as the process of cell death and is an important factor in the development of neurodegenerative diseases since the increase in the formation of ROS and RNS can generate an increase risk; when brain tissue is damaged due to an acute or chronic injury, there is an increase in the response of neuroinflammatory and oxidative stress causing the activation of the immune system with the presence of reactive astrocytes, cerebral parenchyma, and macrophage and microglia cells [27, 43]. MT regulates the expression of inflammatory factors mainly cytokines IL-6, IL-3, and TNF-*α* and interferons which are present in astrocytes [44, 45]; IL-6 is one of the important cytokines upregulated during brain damage, as it increases phagocytosis and the release of inflammatory mediators which in turn induce the site of MT as neuroprotection proteins [43, 44].

Oxidative stress, a condition that occurs due to imbalance between the oxidant and antioxidant status, increases with age and can therefore be considered as a major causative factor in neurodegenerative diseases [46]. Several studies have shown sufficient evidence that oxidative damage plays a vital role in the pathophysiology of neurodegenerative diseases (Alzheimer's disease and Parkinson's disease) and other brain disorders such as cerebral ischemia and epilepsy [47–51]. Increased oxidative stress in brain disorders is often associated with antioxidant defense mechanisms, including MT-I and MT-II; the MT is able to reduce oxidative damage by attenuating the damage caused by oxidative stress, suggesting that the functions of this protein family are more complex and varied than were initially considered [52].

## 6. Alzheimer's Disease

Alzheimer's disease (AD) is the most common cause of dementia, an age-related neurodegenerative disorder, characterized clinically by a progressive deterioration of higher mental functions and cognitive functions, resulting in severe dementia [53]. The neuropathology is characterized by the accumulation of two types of fibrous material in the brain: the A*β*-amyloid extracellular peptide deposited in senile plaques and intraneuronal neurofibrillary tangles (NFT) composed mainly of abnormal and hyperphosphorylated forms of the microtubule-bound tau protein, in addition of the presence of a large number of reactive astrocytes, signs of neuroinflammation, metal dyshomeostasis, and oxidative stress [53–56]. The formation of NTFs and the resulting degeneration first appear in the hippocampus, especially pyramidal neurons in CA1 and layer II of the entorhinal cortex and the neocortex [57]. Several hypotheses have been proposed to explain the pathophysiology of AD, including the amyloid cascade with accumulation of A*β*, the metal ion hypothesis that proposes that the underlying cause of AD is the homeostasis of the deteriorated metal, in particular Zn, Cu, and Fe which leads to amyloid imbalance, oxidative stress leading to mitochondrial dysfunction, metallic ion dyshomeostasis, inflammation, improved apoptosis, and imbalance A*β* [56, 58, 59].

It is commonly accepted that ROS play an important role in the pathogenesis of AD disease with low brain regeneration rate and insufficient antioxidant potential [60–62]; the brain favors oxidative stress, due microglia activation to neuronal lesions which generate excess superoxide radicals [56, 63]. Different reports have suggested that oxidative stress promotes the production of A*β*-amyloid and induces alteration of antioxidant enzymes by increasing levels of oxidative stress generating an increase in A*β* deposition [49, 64, 65]. The A*β*-amyloid peptide present in the astrocytes constituting the senile plaques observed in AD disease is considered a source of free radicals; evidence has shown that A*β*-amyloid enters the mitochondria, and induces the generation of free radicals that promote cross-linking of proteins, through the formation of amyloid-fibril, and Aβ itself can form free radical peptides which can lead to oxidative damage in *in vivo* and *in vitro* neurons [54, 66]. Likewise, experimental studies found that A*β* affects mitochondrial DNA and proteins, which leads to deficiencies in the electronic transport chain (ETC) and, finally, mitochondrial dysfunction [67], thus contributing to the neurodegeneration.

Reactive astrocytes present in patients with AD show significant high levels of MT expression suggesting that MT levels can serve as a marker of AD [68]. The overinduction of MT-I and MT-II in AD stimulated primarily by the presence of free radicals, metal ions, and cytokines may be a defense cellular response against inflammatory signals supporting a neuroprotective effect of MT [3, 56]. Likewise, experimental studies on transgenic animals also have shown that astrocytes and microglia/macrophages surrounding amyloid plaques have high levels of expression of MT-I and MT-II in regions of the cortex and hippocampus [48, 69]. On the other hand, Valko et al. [47] demonstrated that predominantly upregulated MT-I in reactive astrocytes has a neuroprotective effect induced by dual mechanisms in AD, direct attenuation of neurotoxicity of A*β* and indirectly due to MT-I inhibition of A*β*-induced microglial activation and subsequent neurotoxicity.

Another neuropathological feature of AD is the alteration of the Zn metabolism and the accumulation of the metal inside the amyloid plaques. *In vitro* studies have shown that Zn is capable of inducing amyloid A*β* formation, while Cu and Fe only produce partial aggregation [41, 70]. Deibel et al. [71] reported a significant decrease in Cu and significant increases in Zn and Fe levels in the hippocampus and amygdala which are areas that show severe histopathological alterations in patients with AD; due to its role in the homeostatic maintenance of Zn and Cu, the MT is considered an important factor within the pathophysiological mechanisms of the disease. It has also been reported in *in vitro* studies that MT-2A has been able to prevent aggregations of A*β*40 and A*β*42 which are countermediated by preventing neurotoxicity [31].

Expression of MT-III has been observed in different neurological diseases; however, the molecular mechanisms of MT-III downregulation in neurodegenerative disease are not yet known, and unfortunately there are several reports with conflicting results of MT-III function in AD. MT-III is expressed in abundance in normal but largely reduced in AD brains; in *in vitro* studies, an inhibition in neurite formation and survival of cortical neurons has been reported [37, 72, 73]. MT-III and alterations in Zn homeostasis have been correlated with neuronal loss or duration of disease implying increased susceptibility to oxidative stress and metal-induced neurotoxicity [41]. MT-III can bind more metal ions, its role as Zn buffer and sensor/signaling, and also has a periodic response to neuronal insult, while other isoforms are uniformly protective [56]. In contrast to MT-I, the expression of MT-III mRNA was not significantly altered in any of the studied models of AD, probably because MT-III is insensitive to inflammation [48, 69]. MT-III has anti-A*β* activity and antagonizes the neurotoxic effects of A*β* peptides primarily by abolishing the formation of toxic aggregates of A*β* peptides [74]. Decreased MT-III expression in patients with AD suggests a possible decreased protective effect in the AD brain [73].

## 7. Parkinson's Disease

Parkinson's disease (PD) is the most common neurodegenerative disease of the elderly and is a neurological syndrome manifested by the combination of tremor, stiffness, bradykinesia, and loss of postural reflexes; other symptoms known as nonmotor symptoms but associated with the disease are the autonomic, sexual, and sleepy disease which is capable of causing cognitive, psychiatric, autonomic, and sensory disturbances [75, 76]. PD occurs predominantly in the 6th decade of life, and men have a 1.5 to 2 times more likely of contracting disease than women [77]. The neuropathology of this disease is characterized by a gradual and selective loss of dopaminergic neurons in all basal ganglia, causing both dopamine and its metabolites to decrease in the caudate nucleus, putamen, *globus pallidus*, and compact pars of the substantia *nigra* [78].

An important neuropathological feature within the disease is the presence of Lewi bodies in the brain tissue that are intraneuronal filamentous inclusions containing phosphorylated neurofilaments called *α*-synuclein protein [79]. The molecular mechanisms described in PD include damage to striatal neurons by exogenous and endogenous toxin such as free radical, due to deficiencies in mitochondrial function, exitotoxicity, inflammation, and cellular apoptosis, and all these processes eventually result in neurodegeneration. Low levels of antioxidants and high levels of free iron make black matter highly vulnerable to ROS attack, so that oxidative stress has been recognized as an important component of this disease [78]; therefore, it has been proposed that MT may play a role in protecting neurons from oxidative stress damage.

Studies in patients with Parkinson's disease by molecular analysis and immunocytochemical techniques demonstrated an increase in the expression of megalin (neuronal metallothionein receptor) and MT in reactive astrocytes of the substantia *nigra*, supporting the neuroprotective role of MT in these cells [80]. Experimental studies have shown that in advanced Parkinson's disease, dopamine and its metabolites are depleted and damaged dopaminergic neurons can release excess cytosolic dopamine out of the synaptic vesicle, inducing neurotoxicity through the generation of reactive oxygen and dopamine species quinones. In studies performed on hemiparkinsonian models of M-I and MT-II knockout mice, dopamine-doped cells treated with dopamine and L-DOPA injections showed that intrinsic MT protects against neurotoxicity induced by dopamine quinone [81]. On the other hand, studies in 6-hydrodopamine- (6-OHDA-) induced hemiparkinsonian rat models showed that treatment with Levodopa significantly increased MT-III mRNA expression in the *striatum* on the uninjured side, but did not show any significant effect on the injured side with 6-OHDA; these results suggest that regulation in MT-III mRNA expression may be related to progressive degeneration in parkinsonism [82].

Several studies carried out by the Ebadi group have shown in the brain of mice from transgenic and knockout PD models that induction of MT is involved in the synthesis of coenzyme Q10, through the activation of lipoamide dehydrogenase, enhancing the antioxidant capacity of coenzyme Q10 Ubiquinone, by increasing the ubiquinol synthesis, attenuation of *α*-synuclein nitration, as well as suppression of proinflammatory cytokines and TNF*α* [50]. Similarly, *in vivo* and *in vitro* studies examined the peroxynitrite ion generator (ONOO-) 3-morpholinosidnonimine (SIN-1) and observed that the lipid peroxidation induced by SIN-1, reactive oxygen species synthesis, caspase-3, and apoptosis were attenuated by overexpression of the MT gene and increased by MT gene regulation [83]. Coenzyme Q participates in the generation of a proton gradient through the membranes to lead to the formation of ATP; their identification as an important production of antioxidants through cell membranes as well as participation in other redox membrane systems introduces new functions [51], and through these results, the authors have been able to establish dopaminergic neuroprotective mechanisms of MT in PD [83] as well as their new role as a possible biomarker to confirm disease [20].

## 8. Cerebral Ischemia

**Figure 4:**
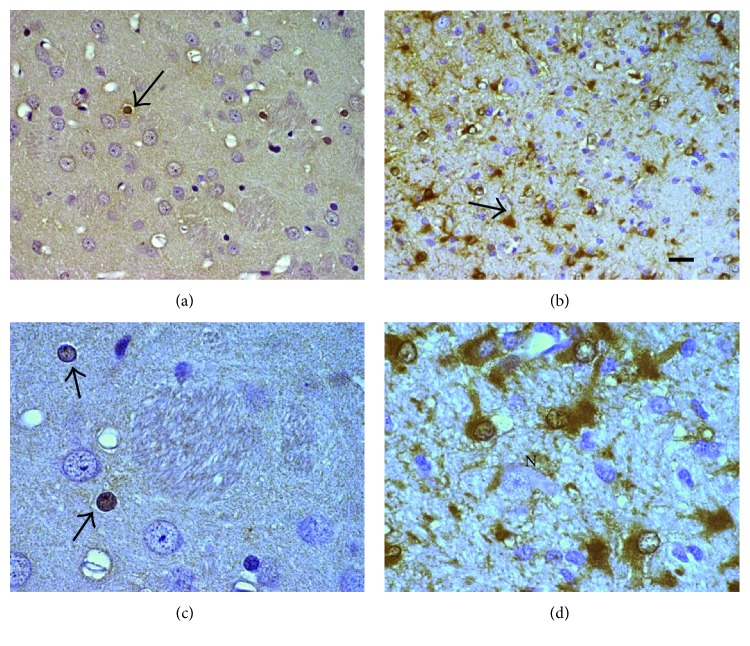
Representative images of immunohistochemical study of MT in the brain (striatum) after MCAO. Control rats (a, c) and MCAO (b, d). (a, c) Neurons, astrocytes, and fibers with normal appearance. (b, d) Interstitial edema, altered neurons (N), and reactive astrocytes are observed after MCAO. Strong immunoreactivity to MT (brown color) was consistently detected in reactive astrocytes (↑) in treated animals. Magnification (a, b) 400x and (c, d) 1000x.

Ischemic stroke is the second leading cause of death worldwide, and the absolute number of stroke patients continues to rise despite a decline in standardized rates of age-related mortality from stroke in the past two decades [84]. Focal cerebral ischemia has been shown to be a passive, acute event, in which energy failure leads to damage in the cerebral tissue causing necrosis and cell death (Figure 4); studies in experimental animals have shown that subsequent events involve expression of genes (including MT) which are related to inflammatory processes and apoptosis and contribute significantly to cell death after the ischemic event [85]. An important factor for cell death after cerebral ischemia is the increase of free intracellular Zn; when ischemic damage produces this accumulation of Zn, it is likely that MT is saturated, and together with the production of hydrogen peroxide, they are able to induce the expression of the MT gene by activating the transcription factor-1 and other promoters of the gene [86]. Transient focal ischemia of 1-2 hours in duration induces an increase in the transcription of certain genes that serve as a protection system against cell damage; the increase gene expression is found in the infarct border area while in the central zones of the cerebral infarction, the processes of transcription and translation of genes are suppressed so that cells die rapidly [87]. Experimental evidence also has reported that after mild cerebral ischemia, there is a rapid induction of MT-I in endothelial cells of cerebral capillaries and astrocytes, showing that it is one of the first detoxification genes in response to damage in brain tissue [88, 89]. Studies in mice with serial gene expression analysis (SAGE) showed that MT-II mRNA was identified as the most significant induced transcript in the early phase of ischemic stroke in the ischemic hemisphere, demonstrating that the maximal concentration of MT occurs at 12 and 24 hours after ischemic damage, expressed almost exclusively in astrocytes of the periphery of the infarcted zone, in the hippocampus, corpus callosum, and striatum regions indicating that the MT gene is overregulated during cerebral ischemia [90].

Although the role of MT-III in the ischemic event remains unknown, some studies have reported an increase in MT-III concentration supporting its neuroprotective role in cerebral ischemia. MT-III mRNA levels were found upregulated in brains after ischemic stress; immunohistochemical analysis showed an increase in immunoreaction in the neurons of the CA1–3 regions of the hippocampus, dentate gyrus, cerebral cortex, olfactory bulb, and Purkinje cells in the cerebellum distributed in the same regions of a normal brain [91]. In knockout mice M-III and wild-type, no significant differences were found after to the 24 h permanent middle cerebral artery occlusion (MCAO). MCAO-induced cerebral infarction in MT-III KO mice was aggravated in comparison to wild-type mice; neurological deficits were found at 5 and 7 days after the cerebral ischemia model. TUNEL tests and markers of oxidative stress were performed on brain tissue and were observed to improve at 24 h after transient MCAO, suggesting that MT-III also has a neuroprotective and antioxidative role in transient cerebral ischemia [92].

The MT-I and MT-II are induced by the inflammatory response generated during cerebral ischemia, mediated by microglial cells, macrophages, and astrocytes with reactive gliosis, which activate proinflammatory mediators that form ROS and oxidative stress that result in the induction of neuronal damage and death [93]. One of the causes of overexpression of MT in ischemia is the association that exists between the factors related to the inflammation process and the response elements which, as mentioned above, are capable of inducing MT expression. It has been reported in *in vitro* and *in vivo* studies that MT promotes the regeneration of neuronal axons as a response to brain damage and that extracellular MT plays an important role in this process and was proposed to the extracellular MT-II as a mediator of its effects on neuronal cells, since through membrane receptors of the low-density lipoprotein family, mostly lipoprotein 1 receptors (megalin). It transfers MT-II from the extracellular medium to the neuronal cytoplasm; this is an important advance in the knowledge of the functions of MT, since extracellular functions of MT do not exclude intracellular functions in damage brains [35, 94, 95].

Diaz-Ruiz et al. [84] reported a protective effect of the application of exogenous MT in rats exposed to events of transient cerebral ischemia and reperfusion; the animals received exogenous MT, and a decrease in lipid peroxidation levels in hippocampus and frontal cortex brain regions as well as a decrease in the area of damaged tissue in the hippocampus after cerebral ischemia was found. According to these results, we observed by immunohistochemical studies in reactive astrocytes a marked increase in the MT immunoreactivity in the peripheral ischemic damage area; the histopathological aspects of the reactive astrocytes and their high immunoreactivity provide evidence of an overexpression of MT in the area of ischemic damage (Figure 4).

On the other hand, several authors have used *in vitro* and *in vivo* models of middle cerebral artery occlusion (MCAO) to analyze the effect of the exogenous application of MT. The results show that there is no protection at 60 min in the MCAO model; however, there was a significant reduction in the volumes of direct and indirect infarction and neurological deficit in the MT-II-treated animal. In addition, MT-II also improved the survival of mice after MCAO, probably due to the suppression of induction of TNF-mRNA in ischemic brain tissue, protecting primary neuronal cells against oxygen-glucose deprivation *in vitro*, supporting the potential therapeutic of exogenous MT administration in experimental stroke models [96, 97].

## 9. Epilepsy

Epilepsy is the most common neurodegenerative disease after stroke, and, according to epidemiological studies, approximately 70–80% of epilepsy patients achieve remission and approximately 30% of these patients are resistant to pharmacological treatment [98]. Epilepsy is characterized by a large number of syndromes that vary between its clinical characteristics, treatment, and prognosis; over time, the various classifications of the disease grouping the seizures (symptoms) and the syndromes of epilepsy have been improving. Epilepsy is a neurological disorder in which it has been suggested that oxidative and nitrosative stress are contributing factors due to prolonged neuronal hyperexcitation and loss of neurons during seizures causing cellular dysfunction. Several studies conducted in humans and experimental animals have demonstrated the relationship between epilepsy and oxidative stress; the generation of free radicals can lead to prolonged seizures that can lead to mitochondrial dysfunction in the hippocampus that precedes neuronal cell death causing subsequent epileptogenesis, although it is not yet known whether oxidative stress is a cause or consequence of this pathology [99, 100].

Dalton et al. [17] were the first to determine that rats treated with kainic acid (KA) as a model of epilepsy showed severe brain damage partly caused by oxidative stress. They found that KA induced an increase in mRNA of MT-I and heme oxygenase-I, as well as the induction of interleukin-1*β* gene expression, triggering an inflammatory response in damaged brain regions, suggesting that oxidative stress produced by KA is able to induce seizures. Since then, experimental models of epilepsy have proven to be useful tools and complementary strategies to advance our understanding of this disease and the molecular mechanisms associated with the role of MT. In the KA model, excessive activation of excitatory glutamate receptors results in sustained epileptic activity; pyramidal neurons of the hippocampus are particularly vulnerable to the neuroexcitatory actions of KA, showing that the vulnerability of neurons to oxidative stress varies from one brain region to another [100, 101].

**Figure 5:**
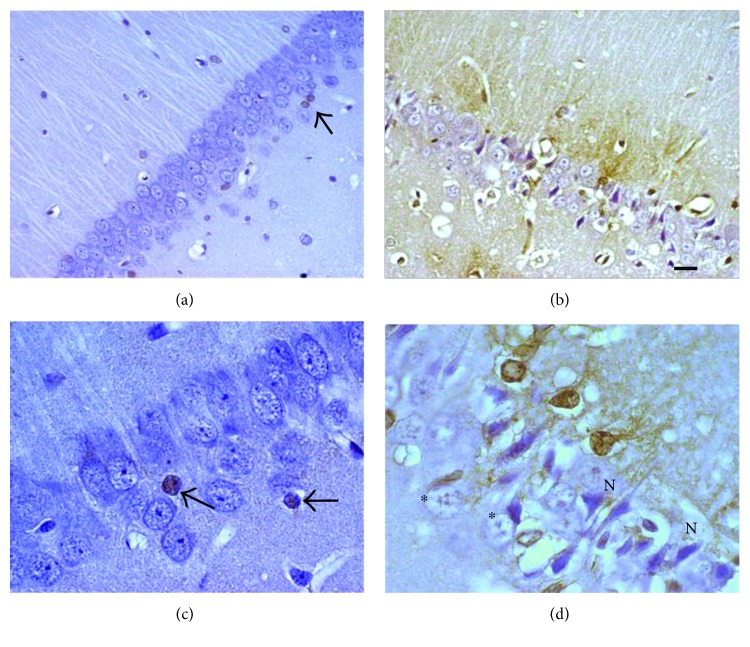
Representative images of the immunohistochemical study of MT in the hippocampus of rats treated with KA. Control rats (a, c) and rats treated with KA (b, d). (a, c) A normal structure of pyramidal cells and astrocytes (↑) in untreated animals. (b, d) Neurons with pyrolysis (∗), pyknotic (N), and neuropil neurons are observed with the presence of interstitial edema in the hippocampus of experimental rats. In the astrocytes (↑) of the rats treated with KA, an increase of the immunoreactivity of the MT (brown color) can be observed. Magnification (a, b) 400x and (c, d) 1000x.

We have previously reported immunohistochemical studies in animals exposed to KA, and an increase in the immunoreactivity of MT in the cytoplasm of astrocytes CA1, CA3, and dentate gyrus of the hippocampus was observed (Figure 5), as well as a significant increase in expression of MT-I and MT-II between 6 and 24 h after KA injection; these results contributed by our group strongly support the evidence that MT plays a major role as an early expression protein in epileptic seizure models [101]. Similar results were observed by Kim et al. [102]. They reported an increase in MT-I and MT-II mRNA expression at 2 and 6 h in the rat brain after treatment with KA, and MT-I appeared to be increased in neuron more evidently in glia, whereas the opposite profile was observed in the case of MT-II; although both MT-I and MT-II expressions showed similar sequential changes (first in neurons and then in glia) after treatment with KA, there is a substantial difference in expression between these two genes. Several studies have clearly demonstrated that high levels of MT-I and MT-II are associated with reduced neuronal death following an induced seizure attack during excitotoxicity. MT-I and MT-II knockout mice show increased levels of oxidative stress, neuronal death, and epileptic seizures after being treated with KA [103]. Likewise, studies carried out on transgenic mice with MT-I overexpression showed reduction of hippocampal inflammation and delayed neuronal degeneration 3 days after exposure to KA; a significant reduction in the proinflammatory response and a delay in the presence of cellular damage were also observed [104]. Other studies with neuronal MT-III have suggested that in special circumstances, the MTs could contribute to the neuronal death; administration of KA in the ventricles also caused a positive regulation of MT-III in reactive astrocytes around the degenerate neurons of the CA3 region of Ammon's horn 3 days after injury, acting as an acute phase protein in reactive astrocytes [105]. Contradictory results have been reported in wild-type mice treated with KA; it was observed that MT-III protects against seizures induced by KA and decreasing neuronal damage likely due to protection against oxidative stress associated with neuronal excitation [106].

On the other hand, similar results have been reported in patients with MTLE. Peixoto-Santos et al. [107] reported that both MTLE and TLE patients increase MT-I and MT-II expressions in all subfields of the hippocampus and in the *dentata fascia* associated with increased astrogliosis; MT-I and MT-II expressions correlate with the astroglial population but not with neuronal population. According to these results, our group reported through a histopathological study that a marked expression of MT-I and MT-II was present in the cytoplasm and neuropile in brain regions of patients with ETL, providing more evidence about the role that MT plays in epilepsy [108].

## 10. Conclusion

This article provides evidence and an overview of the role of MT in normal and pathological conditions in the CNS. Recent research has provided information about the physiological function of MT-I and MT-II in processes of oxidative stress, apoptosis, inflammation, detoxification, and homeostasis of essential metals which are widely related in the pathophysiology of neurodegenerative diseases and other brain disorders. MTs are very important proteins in brain functioning mainly located in astrocytes which can protect neurons against exitotoxicity induced by experimental models of epileptic seizures or in damaged brain tissue as a result of focal cerebral ischemia through modulation of events such as inflammation, oxidative stress, and apoptosis. On the other hand, MT-III found mainly in neurons seems to be involved in the control of amyloid plaque aggregation proteins or *α*-synuclein and neurofilaments, which may lead to the development of neurodegenerative disorders such as Alzheimer's disease and Parkinson's disease. In conclusion, each of the MT isoforms present in the brain may function differently, indicating a multifunctional role over a wide range of cellular processes. However, it is necessary to carry out future studies on the expression and regulation of MT genes to conclusively elucidate and detail the molecular mechanisms involving MT in the nervous system and what their role is in the pathophysiology of brain disorders.
